# Orbital inflammatory disease in a primarily black patient population

**DOI:** 10.3389/fopht.2025.1576929

**Published:** 2025-05-22

**Authors:** Gabriel Kabarriti, Ali Elsayed, Giannis A. Moustafa, Nickisa Hodgson

**Affiliations:** ^1^ Department of Ophthalmology, State University of New York (SUNY) Downstate, Brooklyn, NY, United States; ^2^ Department of Ophthalmology, Kings County New York City Health and Hospitals Corporation (NYC HHC), Brooklyn, NY, United States; ^3^ Department of Ophthalmology, University of California, San Francisco, San Francisco, CA, United States

**Keywords:** orbital inflammation, orbital disease, orbital inflammation treatment review, orbital inflammatory disease, black patient population

## Abstract

**Purpose:**

The purpose of this study is to characterize orbital inflammatory disease (OID) in a primarily Black patient population, examining their demographics, presentations, workup, treatment, and outcome.

**Methods:**

A retrospective study was performed from January 2005 to June 2022 at two academic institutions in Brooklyn, NY. Patients included met criteria for one of the following OID conditions: non-specific orbital inflammation; nonbacterial dacryoadenitis; Tolosa-Hunt; orbital myositis; definite, possible, or probable IgG4-related ophthalmic disease; and sclerosing orbital inflammation. Data reviewed included orbital inflammatory labs, imaging, pathology, and treatment. Treatment was considered successful if a patient had complete resolution of symptoms.

**Results:**

Thirty-nine patients met criteria for this study. 35.9% were diagnosed with dacryoadenitis, 28.2% with NSOI, 12.8% with myositis, 5.1% with possible IgG-ROD, 7.7% with probable IgG4-ROD, 7.7% with Tolosa Hunt, and 2.6% with sclerosing OI. 91% were started on steroids; 12.8% required transition to steroid-sparing therapy. 85% had a successful outcome with a resolution of symptoms.

**Conclusions:**

This study characterizes OID in a Black patient population and compares it to prior studies done on OID. Research on underrepresented patient populations is needed to understand differences in disease presentation and improve patient outcomes.

## Introduction

Orbital inflammatory disease (OID) comprises a heterogeneous group of inflammatory disorders affecting the orbit (1). The classic clinical presentation of OID is described as an acute onset of orbital pain, edema, and proptosis (2). Variations exist in the clinical presentation as OID can affect any orbital structure acutely, sub-acutely, or chronically, making OI challenging to diagnose (3).

Retrospective studies have been conducted with the goal of better delineating OID clinically. In 1968, Blodi and Gass reviewed 140 biopsy-proven OID cases in a predominantly White patient population and found that patients presented with proptosis (57%), diplopia (42%), edema (42%), and pain (24%) (4). In 1996, Gunalp et al. reviewed 132 patients with suspected OID and found that proptosis (82%) was the most common presenting condition, followed by motility restriction (54%) and visual acuity loss (38%) (5). Yuen and Rubin examined 65 patients with suspected OID at Massachusetts Eye and Ear Infirmary and found that pain (69%) and edema (75%) were the most common initial presentations (6). Lee et al. reviewed 141 biopsy-confirmed OID cases across multiple institutions in the US and found that White people were more affected than Black and Hispanic people; the initial presentation was more commonly proptosis (55%) and diplopia (42%) than pain (35%) (7). Young et al. examined 70 biopsy-confirmed OID cases in a primarily Asian population and found that Asian people presented clinically with edema (77%) and erythema (54%) more commonly than pain (33%), proptosis (33%), or diplopia (30%) (8). The clinical variability seen in different groups makes it difficult to create guidelines or classification schemes to guide the clinician in diagnosing OID (9).

The goal of this study is to examine OID in a primarily Black patient population. We sought to conduct an observational study of OID in a Black patient population, examining demographics, presentations, workup, treatment, and outcomes to better characterize this disease in this patient population.

## Methods

A 17-year retrospective study from 2005–2022 was performed reviewing charts of patients diagnosed with OID at two clinical academic sites in Brooklyn, New York. Charts were reviewed for patients with an OID diagnosis or OID subtypes. The subtypes included were based on earlier classifications accepted in the literature as clinicopathologically distinct entities from nonspecific OID. These subtypes were Tolosa-hunt, non-bacterial dacryoadenitis, myositis, IgG4-related ophthalmic disease (ROD), and sclerosing OID. The diagnostic criteria for IgG4- ROD proposed by Goto et al. was used for this study which includes radiologic, histopathologic and laboratory values to determine the probability of IgG4-ROD; *definite ROD* indicates having radiologic, histopathologic and laboratory findings, *possible ROD* indicates having radiologic and histopathologic findings, and *probable ROD* indicates having radiologic and laboratory findings (10). The pediatric (aged 0-18) and adult population (aged 18+) were included if they self-identified as Black or African American people. An institutional HIPAA waiver was obtained before initiating the study. Institutional review board approval was obtained.

Data collected included patient demographics, comorbid ocular disease, comorbid autoimmune conditions, initial and final comprehensive ophthalmologic exam findings, laboratory results, imaging, histological findings, management, outcomes, and recurrence rates. Laboratory markers included general non-specific markers (CBC, ESR, CRP), infectious (HIV, RPR, EBV), and autoimmune (ANA, RF, IgG4, ACE, lysozyme, c-ANCA, p-ANCA, SS-A, SS-b, IgE).

Treatment response was considered successful if a patient experienced a complete resolution of symptoms with no associated pain, extra-ocular muscles (EOM) restriction, or evidence of inflammation.

## Results

Thirty-nine patients met criteria for this study. Demographics were listed in **Table 1**. 69.2% were females. 71.8% of patients were younger than 50 years of age with a median age of 35 years. 7.7% of patients presented with a prior history of OID diagnosed at outside institutions.

**Table 1:** Demographics and clinical characteristic of patients with OID.

	Total, n (%)^a^	Dacryoadenitis, n (%)^a^	Orbital myositis, n (%)^a^	IgG4-Probable disease, n (%)^a^	IgG4-possible disease, n (%)^a^	Tolosa-Hunt syndrome, n (%)^a^	NSOI, n (%)^a^	Sclerosing OI, n (%)^a^
Total	39	14	5	3	2	3	11	1
Age, years								
<25	11 (28)	6 (43)	1 (20)	1 (33)	0 (0)	0 (0)	3 (27)	0 (0)
25-49	17 (44)	4 (29)	3 (60)	1 (33)	2 (100)	1 (33)	5 (45)	1 (100)
≥50	11 (28)	4 (29)	1 (20)	1 (33)	0 (0)	2 (66)	3 (27)	0 (0)
Gender								
Female	27 (69)	11 (79)	4 (80)	2 (67)	1 (50)	3 (100)	5 (54)	0 (0)
Male	12 (31)	3 (21)	1 (20)	1 (3)	1 (50)	0 (0)	6 (46)	1 (100)
Autoimmune disease								
Present	2 (5)	2 (14)	0 (0)	0 (0)	0 (0)	0 (0)	0 (0)	0(0)
Not present	37 (95)	12 (86)	5 (100)	3 (100)	2 (100)	3 (100)	11 (100)	1(100)
Symptom onset to presentation, days								
<7	23 (59)	10 (71)	3 (60)	2 (67)	0 (0)	1 (33)	5 (46)	1 (100)
Jul-29	8 (21)	2 (14)	1 (20)	1 (33)	0 (0)	1 (33)	3 (27)	0 (0)
≥30	8 (21)	2 (14)	1 (20)	0 (0)	2 (100)	1 (33)	3 (27)	0 (0)

NSOI, nonspecific orbital inflammation.

a Percentages were rounded up to integer numbers and may not add up to 100%.

49% of patients presented within one week of symptom onset. 28% presented within 7 to 29 days, and 23% presented after 30 days of symptom onset. **Table 2** showed the distribution of symptoms. The most common presenting symptoms were pain (64%) and edema (59%). 87% had unilateral disease with the right eye affected in 51% of patients and the left affected in 36% of patients. 13% had bilateral disease. Most patients (90%) presented with vision of 20/40 or better, while 10% of patients had vision worse than 20/40.

**Table 2:** Presenting symptoms of OID.

Presenting Symptom	Number of patients (n), percent
Pain	25, (64%)
Edema	23, (59%)
Redness	5, (13%)
Ptosis	4, (10%)
Discharge	3, (7%)
Diplopia	3, (7%)
Tearing	2, (5%)
Chemosis	2, (5%)
Blurry Vision	2, (5%)
Proptosis	2, (5%)

The final diagnosis and classification of OID in this group were as follows: 35.9% (14/39) with dacryoadenitis, 28.2% (11/39) with NSOI, 12.8% (5/39) with myositis, 5.1% (2/39) with possible IgG-ROD, 7.7% (3/39) with probable IgG4-ROD, 7.7% (3/39) with Tolosa Hunt, and 2.6% (1/39) with sclerosing OI.

Laboratory markers were collected. Of all 39 patients, 35/39 (90%) had a complete blood count, of which only 1/35 (2.8%) had an elevated WBC greater than 11,000/microliter. 17/39 (44%) were tested for CRP, of which 8/17 (47%) had levels above 10mg/l. ESR was tested in 25/39 (64%) and was considered elevated in 15/25 (60%). Of the 14 patients diagnosed with dacryoadenitis, EBV IgG was positive in 4 of 4 patients tested, while EBV IgM was positive in one patient. Patients with positive EBV antibodies were younger with age range 21-41. Of the 14 patients diagnosed with dacryoadenitis, ACE was positive in 2 of 13 (15.3%) patients tested, lysozyme in 1 of 12 (8.3%) patients tested, SS-A in 1 of 5 (20%) patients tested, and C-ANCA 1 of 10 (10%) patients tested.

Of the 5 patients diagnosed with a variation of IgG4-ROD, two diagnosed with possible IgG4-ROD had normal laboratory levels of IgG4 titers, while the three diagnosed with probable IgG4-ROD had evidence of elevated IgG4 greater than 135 mg/dl. Other labs collected SS-B, P-ANCA, rheumatoid factor, TSH, and TSI were negative in all patients.

The remainder of the clinical course is described in **Table 3**. Radiological imaging was performed in 37 patients (94.9%). Computed Tomography (CT) orbits was performed in 66.67% of total patients, MRI in 20.5%, and 5% received both. Enhancement of the lacrimal gland and enlargement was seen in 46.1% of patients. 43.9% had evidence of enlargement of extraocular muscle(s). Of those diagnosed with IgG4-ROD, 2 of 5 (40%) had bilateral lacrimal gland enlargement, one (20%) had unilateral lacrimal gland enlargement, one (20%) had evidence of EOM enlargement, while the last (20%) had evidence of diffuse enlargement of orbital tissue. Of the five patients diagnosed with myositis, 3 (60%) patients had evidence of enlargement of two or more EOMs. 11 of 11 (100%) patients diagnosed with NSOI had evidence of inflammatory changes throughout the orbit without specific localization. Chest x-ray was performed in 49% of patients and 2.7% had findings concerning for a calcified granuloma in the chest.

**Table 3:** Management and outcomes of patients with OID.

	Total, n (%)^a^	Dacryoadenitis, n (%)^a^	Orbital myositis, n (%)^a^	IgG4-Probable disease, n (%)^a^	IgG4-possible disease, n (%)^a^	Tolosa-Hunt syndrome, n (%)^a^	NSOI, n (%)^a^	Sclerosing OI, n (%)^a^
Total	39	14	5	3	2	3	11	1
Treatment								
Steroids Only	21(53)	8 (57)	3 (60)	1 (33)	0 (0)	2 (66)	5 (45)	1(100)
Steroids + Antibiotic	15 (38)	4 (29)	2 (40)	2 (67)	2 (100)	0 (0)	6 (55)	0 (0)
NSAIDs	1 (2.6)	1 (7)	0 (0)	0 (0)	0 (0)	0 (0)	0 (0)	0 (0)
No treatment	1 (2.6)	0 (0)	0 (0)	0 (0)	0 (0)	1 (33)	0 (0)	0 (0)
Refused treatment	1 (2.6)	1 (7)	0 (0)	0 (0)	0 (0)	0 (0)	0 (0)	0 (0)
Imaging								
Performed	37 (95)	13 (93)	5 (100)	3 (100)	2 (100)	3 (100)	10 (91)	1 (100)
No Imaging	2 (5)	1 (7)	0 (0)	0 (0)	0 (0)	0 (0)	1 (9)	0 (0)
Biopsy								
Performed	14 (36)	3 (21)	0 (0)	2 (67)	2 (100)	1 (33)	5 (45)	1 (100)
No Biopsy	25 (64)	11 (79)	5 (100)	1 (33)	0 (0)	2 (66)	6 (55)	0 (0)
Immunomodulatory agents								
Prescribed	5 (13)	2 (14)	0 (0)	0 (0)	0 (0)	0 (0)	2 (18)	1 (100)
Not prescribed	34 (87)	12 (86)	5 (100)	3 (100)	2 (100)	3 (100)	9 (82)	0 (0)
Outcome								
Successful	33 (85)	12 (86)	4 (80)	3 (100)	2 (100)	2 (67)	9 (82)	1(100)
Unsuccessful	6 (15)	2 (14)	1 (20)	0 (0)	0 (0)	1 (33)	2(18)	0 (0)

NSOI, nonspecific orbital inflammation.

a Percentages were rounded up to integer numbers and may not add up to 100%.

Biopsies were performed on 14 out of 39 patients (36%). Non-necrotizing granulomatous inflammation was present in 2 out of the 14 biopsies (14%). The biopsy results were split amongst probable IgG4-related disease (2/14, 14.3%), possible IgG4-related disease (2/14, 14.3%), non-specific orbital inflammation (5/14, 21.4%), dacryoadenitis (3/14, 21.4%), and (1/14, 7.1%) sclerosing orbital inflammation. One patient with Tolosa Hunt had a biopsy to rule out Giant Cell Arteritis (1/14, 7.1%).

The mainstay of treatment used was systemic immunosuppression. 53% were started on steroids only. 2.6% were treated with non-steroidal anti-inflammatory drugs alone, 2.6% refused treatment, and 2.6% of patients improved without intervention. 38% (15/39) had a course of simultaneous antibiotics and steroids. Of the patients started on dual antibiotic and steroid treatment, 7.7% (3/39) had antibiotics discontinued as infectious causes were excluded.

Symptoms recurred in 28.2% (11/39) of patients. Four of these patients developed symptoms after abruptly stopping medications against recommendations and these patients were lost to follow up after; notably 3/4 of these patients were diagnosed with myositis. 4/11 developed symptoms while tapering medication and needed to be restarted on their initial dose of prednisone; they were maintained on methotrexate after steroid taper. Notably, of the four patients started on methotrexate, 2 were diagnosed with NSOI, 1 with sclerosing OI, and 1 with dacryoadenitis. 3/11 developed symptoms after a complete taper of steroids; these three patients, of which 1 diagnosed with Tolosa hunt, 1 with NSOI and 1 with dacryoadenitis, were re-started on prednisone and had complete resolution of symptoms with no evidence of recurrence after taper.

Referrals were made to other specialties for co-management. 23% (9/39) of patients were referred to rheumatology. Of these patients, 5/9 (55%) were started on methotrexate for disease management; 4 of the 5 started on methotrexate were patients who developed recurrence of symptoms after slow taper of steroids. Of the three patients diagnosed with Tolosa Hunt, 2 of 3 were referred to Neurology and Neurosurgery.

85% of patients had successful outcomes with resolution of EOM limitations and extra-ocular pain. 90% of patients had stable vision at 20/40 or better, 2.6% of patients had vision that improved to 20/40 or better, and 7.2% had improved vision from their initial presentation but never reached 20/40 or better. Those maintained on methotrexate had successful resolution of symptoms.

## Discussion

This study is the first attempt to characterize OID in a primarily Black patient population. Compared to other studies, our cohort demonstrated a similar demographic distribution, with females being more affected than males, and the age of diagnosis frequently occurring under 50. Pain and edema were the most common presenting symptoms. Additionally, our finding that OID had a unilateral predilection, aligns with previous research (3, 6, 11).

In this study cohort, females were more likely to be affected than males with a near 2 to 1 ratio, similar to previous research (6, 7). Mombarts et al. suggested that autoimmunity can be an etiology of OID and found that 10% of cases in their study had a concomitant autoimmune condition (12). In this study, a concurrent autoimmune condition was found in 5% of cases. With advances in gene sequencing and studies identifying different gene expression with OID (13), it will remain to be seen whether those identified genes can be found in the Black patient population.

When OID was suspected, a thorough work-up was performed to rule out other etiologies. Laboratory markers helped reveal underlying etiologies of dacryoadenitis and identify cases that were *probable IgG4-ROD.* Inflammatory serum markers, namely ESR and CRP, were elevated in nearly half of patients collected. While these markers were non-specific, it has been suggested that CRP levels greater than 20.2 mg/l had a 90.9% sensitivity and 90.5% specificity in diagnosing orbital cellulitis when differentiating from NSOI in cases of uncertainty (14).

Orbital imaging was critical in helping localize tissue involvement. Lacrimal gland enlargement was the most common imaging finding followed by enlargement of muscles. Lacrimal gland enlargement was associated largely with dacryoadenitis and IgG4-ROD. IgG4-ROD had evidence of bilateral enlargement, consistent with IgG4-ROD being a systemic disease. Enlargement of muscles was associated with multiple diagnoses including IgG4-ROD, NSOI, and myositis. Those diagnosed with myositis had evidence of multiple muscles enlarged. This finding is consistent with the Asian population where multiple muscles were enlarged (8). With a growing knowledge of OID, certain radiological patterns of OID may become more evident across different populations (15).

The decision to biopsy in OID syndrome remains controversial and varies according to the clinician and case. Gunlap et al. recommended that biopsy is safe and reliable once the acute phase of inflammation resolves (5). Young et al. reinforced this decision to confirm histopathologic subtyping (8). A group of international panelists who convened in 2016 recommended that myositic NSOI should first have a course of steroids, while those with non-myositic NSOI should proceed with biopsy (16). In a review of orbital biopsies performed in patients with thyroid-eye disease, NSOI, granulomatosis with polyangiitis, and sarcoidosis, patients diagnosed with NSOI had histopathologic evidence of inflammation, yet the amount of inflammation varied from specimen to specimen. This fluctuation could be attributed to the concomitant use of steroids which may have reduced the inflammatory response (17). In practice, Yuen et al. biopsied 29% of patients suspected with OID who either presented with an atypical presentation or failed initial therapy. In our cohort, 36% percent were biopsied, of which 64% of those biopsies helped reveal a subtype of OID, which otherwise would not have been properly diagnosed. Biopsy remains an important factor in confirming the diagnosis and subtype of OID.

Steroids remain the conventional first-line treatment for OID (18, 19). Sclerosing OI other cases refractory to steroids may require alternative therapy (11, 18, 20). Options for steroid sparing therapy include biologics and radiation therapy. In our cohort, 56% were started on steroids alone, and 49% of patients had an adequate response with the resolution of symptoms. 36% of our cohort underwent co-treatment with antibiotics alongside prednisone, as infectious etiology could not initially be ruled out. Interestingly, five patients (12.8%) who required transition to a steroid-sparing agent methotrexate had a complete resolution of symptoms. Having a complete resolution of symptoms after starting methotrexate contrasts slightly from the white population. Smith et al. characterized the clinical utility of methotrexate in 14 white patients with non-infectious orbital disease, of which included 7 patients were diagnosed with NSOI. Of the 14 patients, 10 patients completed an adequate 4-month treatment trial, while 4 discontinued methotrexate early. Of the 10 patient that did complete methotrexate, 90% had a favorable clinical response; the 1 patient who showed no clinical response was diagnosed with NSOI (21). It would be interesting to see if this remains true with studies involving larger patient populations. In total, 82% of our cohort had resolution of symptoms and improvement in EOM movements and pain. This finding is similar to the study by Yuen et al, which had a complete resolution of symptoms in 63% and were treated with a combination of steroids, radiotherapy, non-steroidal anti-inflammatory agents, or surgical debulking.

This study has its limitations. As a retrospective observational study, it is susceptible to incomplete documentation, missing charts, unrecoverable information, and variability in the information recorded by medical professionals. This study sheds light on the presentation and disease course of orbital inflammatory syndrome in a small Black patient population.

## Data Availability

The raw data supporting the conclusions of this article will be made available by the authors, without undue reservation.
